# Preoperative Evaluation of V-Y Flap Design Based on Computer-Aided Analysis

**DOI:** 10.1155/2020/8723571

**Published:** 2020-01-11

**Authors:** Zong-Lin Yang, Yu-Hui Peng, Cheng Yang, Bo Cheng, Ming-Kai Ji, Yan Zhao

**Affiliations:** ^1^School of Mechanical Engineering and Automation, Fuzhou University, Fuzhou 350108, China; ^2^The Fist Affiliated Hospital of Fujian Medical University, Fuzhou 350009, China

## Abstract

V-Y flap is widely used in plastic surgery as an important technique for reconstructing deformities and improving appearance. In this paper, a geometrical parameter model and finite element analysis were used to study the rationale of the proposed V-Y flap design and the preoperative evaluation of the V-Y flap design. First, a geometric parameter model of the V-Y flap was established to analyze the five key geometric relationships affecting the flap structure and obtain a reasonable plan for the V-Y flap design through the crossing constraint relationship. Second, in order to verify the effectiveness of the V-Y flap design, the suture and release states of the V-Y flap during surgery were evaluated based on a simulation model of the V-Y flap generated by finite element analysis software. The results revealed that the approach proposed in this paper provides a feasible method for clinical V-Y flap design.

## 1. Introduction

As a barrier between the body and the external environment, human skin has an important role in protecting our body, regulating body temperature and sensing external stimuli. Once the skin is damaged, it must be repaired as soon as possible. Flaps are widely used in skin and plastic surgery because they can repair and reconstruct damaged skin [1–4]. The V-Y flap, which consists of an island-like skin with subcutaneous tissue connected to it, has been widely utilized in clinical trials as a local flap. In this approach, no rotation or deformation occurs during surgery and the site remains smooth [5,6]. The incision scar line for the V-Y flap is similar to a triangular kite, and hence this is also known as a “Kite” flap. However, the design and preoperative evaluation of the V-Y flap surgical approach currently rely entirely on the doctor's experience. Therefore, it is difficult to achieve a perfect design proposal for an individual before surgery by relying on past clinical experience. Due to irregular surface morphology, flap retraction, and other factors, the flap design can become more complicated. If the flap is not properly designed, it generally leads to dysfunction. Therefore, experience-dependent flap design has been frustrating for dermatologists and plastic surgeons. As expected, the development of an effective method to predict the postoperative effect and accurately guide the design of V-Y flaps is highly desirable.

The finite element method (FE) is a numerical calculation approach to solve complex mathematical problems, such as physical structure, material properties, and load changes according to the mixed variational principle. Through the combination of medicine and engineering mechanics, computer-aided engineering approaches like finite element analysis have been gradually applied in the medical field. Yin et al. [7] demonstrated the performance of different implant designs from a multiscale biomechanical perspective using the finite element method. Kang et al. [8] applied the finite element method to study the suitability of metal block augmentation for large uncontained bone defects from a biomechanical perspective. Francesca et al. [9] investigated the effects of two different mask designs on the facial tension of subjects' facial skin by the finite element method. Tepole et al. [10] analyzed the relationship between the maximum stress region and tissue necrosis by establishing a finite element model. Retel et al. [11] observed the stress field of a diamond-shaped wound suture by constructing a finite element model to simulate the mechanical behavior of human skin during wound closure. Pauchot et al. [12,13] proposed a simple mathematical model for V-Y flaps and verified the role of skin tension in patients treated with V-Y flaps using finite element analysis. Mi et al. [14] studied the thermal effect of high-voltage nanosecond pulse groups in tumor tissue by parameter analysis based on the finite element model in melanoma tumors. However, very few of the reports have been published to effectively evaluate the rationale of flap design.

In this study, a geometric parameter model of the V-Y flap was proposed and the five key geometric relationships affecting the structure of V-Y flaps, combined with a summary of clinical experience, were addressed. Then, the ideal V-Y flap design was determined by the suggested cross-constraint relationship. Next, a simulated V-Y flap model was constructed using finite element analysis software. As a result, the force, suture, and release states of the flap during V-Y flap surgery were simulated and analyzed to predict the postoperative effect and the effectiveness of the V-Y flap design method proposed was verified.

## 2. Materials and Methods

### 2.1. Geometric Model of V-Y Flap

**Figure 1:**
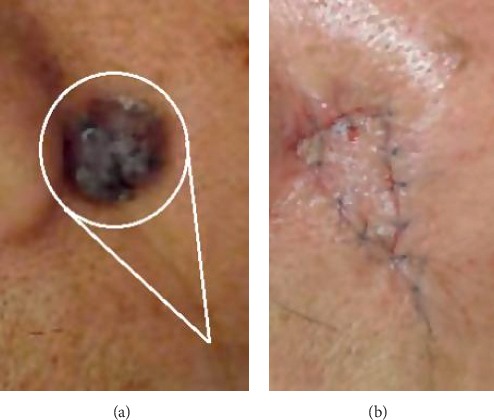
V-Y flap. (a) Preoperative. (b) Postoperative.

**Figure 2:**
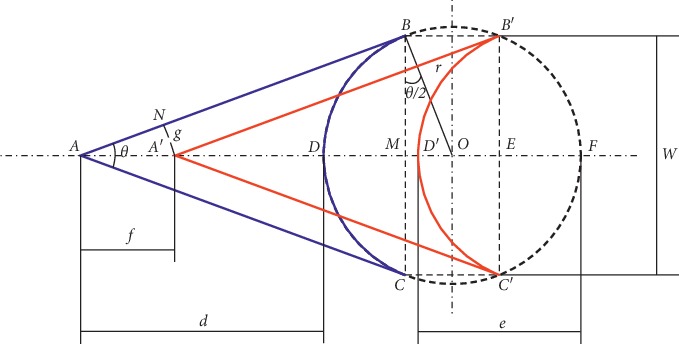
Geometric model of a V-Y flap.

The typical preoperative marking and suturing of a V-Y flap are displayed in Figures 1(a) and 1(b), respectively, and the corresponding geometric V-Y flap model was established as shown in Figure 2. The black dotted circle shows the tumor incision area and the area enclosed by the BC arc, and lines AB and AC represent the local flap. The curve enclosed by the *B*′*C*′ arc and lines *A*′*B*′ and *A*′*C*′ denotes the advancement position of the flap transplant. Considering the symmetry characteristics of the V-Y flap geometry, the upper part of V-Y flap is focused. The parameters of V-Y flap geometry are depicted in Figure 2, where *d* is the length of flap AD and *w* is the width of flap BC.

### 2.2. Key Geometric Parameters of Flaps

#### 2.2.1. Area Ratio of V-Y Flap and Defect *n*

The area of a V-Y flap should not only have a proper ratio with the defect area but also be suitable for suturing. Although a larger flap area may help to improve the success rate of the operation, it will increase the scar area, reducing the postoperative aesthetic effect. However, a smaller flap area may result in too much tension during suturing, which may cause negative effects, such as tissue necrosis in the postoperative period. Therefore, the area ratio of the V-Y flap to the defect plays an important role in flap design.

The area ratio of the flap to the defect is defined as *n*:

(1) $$\begin{array}{c} n=\frac{S_{f}}{S_{w}}, \end{array}$$

(2) $$\begin{array}{c} S_{f}=S_{\Delta ABO}-S_{\text{sector}BDO}, \end{array}$$

(3) $$\begin{array}{c} S_{\Delta ABO}=\frac{1}{2}\;r^{2}\cot\frac{\theta }{2}, \end{array}$$

(4) $$\begin{array}{c} S_{\text{sector}BDO}=\frac{r^{2}}{2}\left(\frac{\pi }{2}-\frac{\theta }{2}\right), \end{array}$$

where *S*_*f*_ is the flap area and *S*_*w*_ is the incision area.

Putting equations (3) and (4) into equation (2):

(5) $$\begin{array}{c} S_{f}=\frac{r^{2}}{2}\left(\cot\frac{\theta }{2}-\frac{\pi }{2}+\frac{\theta }{2}\right), \end{array}$$

(6) $$\begin{array}{c} S_{W}=\frac{\pi r^{2}}{2}. \end{array}$$

Putting equations (5) and (6) into equation (1):

(7) $$\begin{array}{c} n=\frac{\cot\left(\theta /2\right)-\left(\pi /2\right)+\left(\theta /2\right)}{\pi }. \end{array}$$

#### 2.2.2. Flap Length *d*

The sides of a V-Y flap are tangent to the edge of the circular defect:

(8) $$\begin{array}{c} d=AD=AO-DO,\\ AO=\frac{r}{\sin\left(\theta /2\right)},\\ d=r\left(\frac{1}{\sin\left(\theta /2\right)}-1\right). \end{array}$$

#### 2.2.3. Ratio of Flap Length to Flap Width *d*/*w*

The ratio of flap length to flap width determines the geometry of a V-Y flap and is another important parameter in flap design:

(9) $$\begin{array}{c} w=BC=2r\;\cos\frac{\theta }{2},\\ \frac{d}{w}=\frac{\left(1/\sin\left(\theta /2\right)\right)-1}{2\;\cos\left(\theta /2\right)}. \end{array}$$

#### 2.2.4. Ratio of the Side Stitching Width to the Flap Advancement Distance *g*/*f*

A close relationship between the flap suturing size and the apex angle of the flap has been reported [15]. If the side suturing width (*g*) is too large, the postoperative tension inevitably increases, which generally leads to tissue necrosis and worsens the surgical effect. Furthermore, during the process of V-Y flap advancement, the subcutaneous pedicle tissue remains adhered and flap advancement (*f*) is limited to a certain extent:

(10) $$\begin{array}{c} \frac{g}{f}=\sin\left(\frac{\theta }{2}\right). \end{array}$$

#### 2.2.5. Suturing Width of the Bottom Edge *e*

(11) $$\begin{array}{c} e=DF-DD^{\prime },\\ DD^{\prime }=BB^{\prime }=2r\;\sin\frac{\theta }{2},\\ e=2r\left(1-\sin\frac{\theta }{2}\right). \end{array}$$

### 2.3. V-Y Flap Design Based on Crossing Constraints

**Figure 3:**
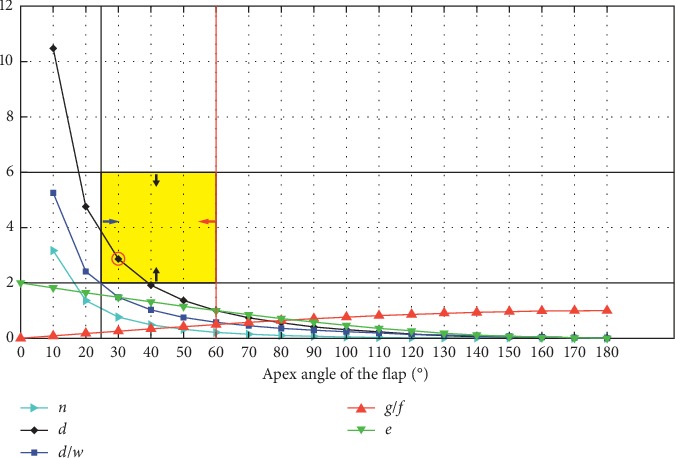
The cross-constraint relationship of V-Y flap geometry.

According to the above geometric parameters of the V-Y flap model, it is apparent that both the defect radius *r* and the flap apex angle *θ* play critical roles in V-Y flap design. By summarizing the published classical clinical experience, the cross-constraints of the V-Y flap geometric parameters were obtained (Figure 3). A defect radius of *r* = 1 cm was assumed.

1. Double-side suturing width (2*g*) should be as less as possible compared to the flap advancement *f*. When the angle *α* ≥ 60° and *g* /*f* > 0.5, it is not suitable for suturing the flap tail [15]. Therefore, a flap apex angle *θ* less than 60° is preferable.
2. The flap length *d* becomes smaller as the angle *θ* increases. *d* decreases sharply as *θ* increases from 10° to 20°. The maximum flap length usually ranges from one to three times that of the defect diameter [16]. Thus, the flap length should be 2 ≤ *d* ≤ 6 cm.
3. The ratio of flap length to flap width gradually decreases with increases in angle *θ*. In order to prevent blood circulation disorders or necrosis at the distal end of the flap, the length-to-width ratio of any flap should not exceed 2 : 1 [17].
4. The area ratio of the flap to the incision *n* decreases with increases in angle *θ*. When 10° ≤ *θ* ≤ 30°, the area ratio *n* decreases sharply, then levels off (Figure 3).

Figure 3 describes the cross-constraint results of the flap geometric parameters. When the apex angle of the flap *θ* equals 30, each geometric parameter summarized from the previous clinical experience yields a feasible value. Thereby, the acceptable flap design is achieved at *d* = 2.86, *n* = 0.77, and *d*/*w* = 1.48.

### 2.4. Finite Element Analysis of V-Y Flap

When the V-Y partial flap is advanced, skin tissue undergoes mechanical creep and elastic stretching by external force with large deformation and large displacement, which enable the defect to be sutured. The optimal V-Y flap design was obtained through the aforementioned geometric model of V-Y flaps. In order to better evaluate the preoperative design of a V-Y flap, a finite element model of a V-Y flap was established. Then, the distribution of suture tension was analyzed and the simulated results verified the feasibility of the V-Y flap design proposed.

#### 2.4.1. Finite Element Model of a V-Y Flap

**Figure 4:**
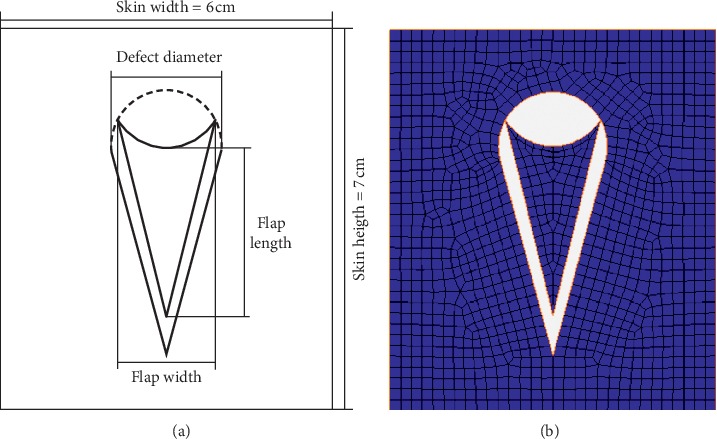
V-Y flap whole model. (a) Schematic diagram of V-Y flap model. (b) Finite element mesh.

Skin thickness is small compared to the skin size, so a skin model can be constructed as a planar two-dimensional model. A 6  cm × 7  cm rectangular skin area model was established with MSC.Marc/Mentat software. The length and width of the flap were set at 2.86 cm and 1.93 cm, respectively, and the apex angle of the flap was 30°. In clinical practice, the stitching interval is about 3∼4 mm [18], so a 3 mm stitching interval was selected. A four-node quadrilateral plane strain unit was applied in the finite element model. Thus, a total of 1260 nodes and 1139 quadrilateral elements were meshed (Figure 4).

#### 2.4.2. Boundary Conditions

During the process of flap suturing, due to the mutual pulling effect, both the flap and the defect undergo certain displacement and deformation. To truly simulate flap advancement, the four corners of the skin area are defined as fully constrained and the edges of the skin model are not imposed any constraints on in this study. Considering the actual surgical process of flap advancement, this study focused on the two corresponding states, suturing and release:

1. Suturing: clinically, after the flap is advanced to a certain position before suturing, an artificial external load is applied to the defect skin edge and the flap. Therefore, the edges of the defect and flap are stretched and deformed. In the FE model, a fixed displacement constraint was applied to the corresponding nodes on the edges of both the defect and the flap. The two parts were displaced relative to each other to simulate the suturing state of the V-Y flap.
2. Release: clinically, the external load is removed after suturing; in other words, the node displacement constraint is removed in the FE model. At this time, the edges of the defect and flap are still adhered to each other. Due to the nonlinear hyperelastic characteristics of skin, the defect and flap produce some internal contraction. The release state attempted to simulate the actual stress and displacement of the V-Y flap after removing the displacement constraints on all suture nodes.

#### 2.4.3. Material Properties

In general, skin is considered to be a kind of nonlinear hyperelastic material. In this paper, the Ogden model was selected for evaluating the properties of the material. The strain energy function of the Ogden model is expressed in the following form:

(12) $$\begin{array}{c} W=\overset{N}{\underset{n=1}{\sum }}\frac{\mu_{n}}{\alpha_{n}}\left[J^{-\alpha_{n}/3}\left(\lambda_{1}^{\alpha_{n}}+\lambda_{2}^{\alpha_{n}}+\lambda_{3}^{\alpha_{n}}\right)\right]-3+4.5K{\left(J^{1/3}-1\right)}^{2}, \end{array}$$

where *J* is the volumetric ratio and equals to *λ*_1_*λ*_2_*λ*_3_ and *λ*_*i*_ (*i* = 1, 2, 3) means the principal stretch ratios; *α*_*n*_ denote the exponential parameters; *μ*_*n*_ are the shear parameters; *K* is the initial bulk modulus; and *N* is the number of the terms.

It is generally recognized that the skin compressibility is small [19], so in this study, the skin material is assumed as incompressible soft biological tissues [20]. For the incompressible material, *J* equals to 1 and the strain energy function also can be transformed as follows:

(13) $$\begin{array}{c} W=\overset{N}{\underset{n=1}{\sum }}\frac{\mu_{n}}{\alpha_{n}}\left[\left(\lambda_{1}^{\alpha_{n}}+\lambda_{2}^{\alpha_{n}}+\lambda_{3}^{\alpha_{n}}\right)\right]-3. \end{array}$$

The parameters' value is listed in the following: *J* = 1, *N* = 2, *μ*_1_ = 6.1244*e* − 7 MPa, *μ*_2_ = 0.1434 MP; *α*_1_ = 3.9246, *α*_2_ = 12.6207.

## 3. Simulation Results

Equivalent von Mises stress criteria were utilized in the output of finite element analysis, and the stress and displacement results are presented in a color cloud diagram.

### 3.1. Suturing State

**Figure 5:**
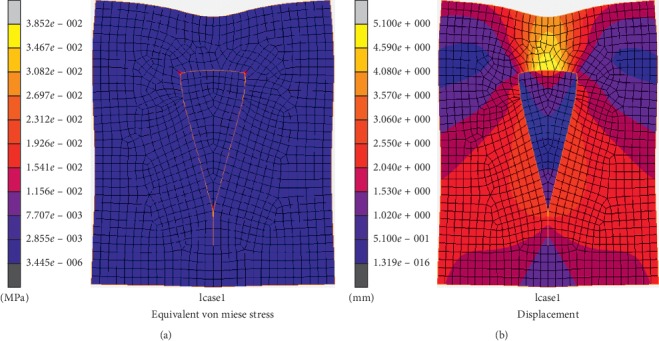
V-Y flap at suturing state. (a) Stress distribution (MPa). (b) Displacement distribution (mm).

The overall von Mises stress distribution of the flap model is displayed in Figure 5(a). The stress concentration of the flap is revealed at the three corner points, which include the apex angle, the top left, and the right end positions, which are consistent with actual clinical practice.

Figure 5(b) shows the overall displacement distribution of the V-Y flap model. The shape of the flap and defect in this state resemble a “kite,” which is consistent with the actual clinical deformation. The colors show the magnitude of the node displacement, where the maximum displacement occurs at the stitching bottom edge of flap and defect (yellow area).

### 3.2. Release State

**Figure 6:**
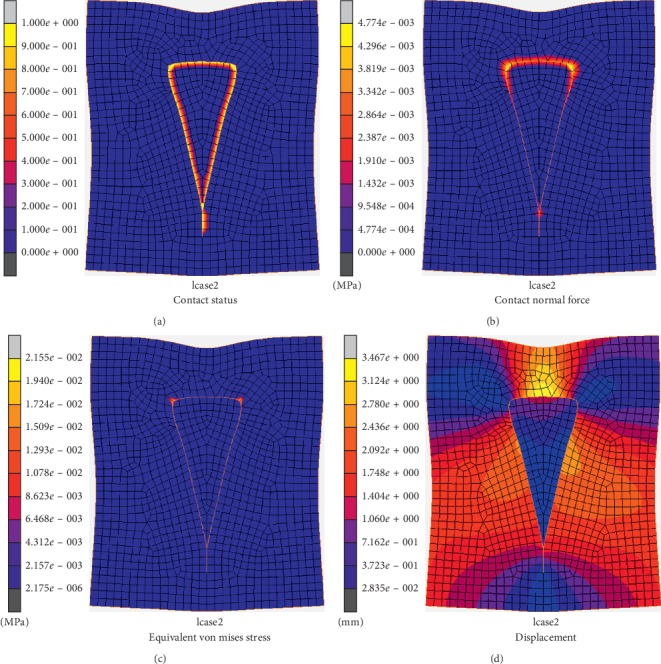
V-Y flap at release state. (a) Contact status. (b) Tension distribution (MPa). (c) Stress distribution (MPa). (d) Displacement distribution (mm).

Figure 6(a) demonstrates the contact conditions of the skin edge and the flap at release state by different colors. The distribution of color along the suturing edges of the flap and defect remains uniform, which denotes no gaps or deformation occurs after removal of the restraints. The skin model obtained a good suturing effect, which reflects a satisfactory flap design rationale.

The stress distribution of the stitching edges is depicted in Figure 6(b). Maximum tension is applied at the flap bottom and the apical angle, which greatly influence the postoperative effect. Therefore, both the suturing width at the bottom and the sides play critical roles in flap geometric modeling.  Figure 6(c) shows the distribution of von Mises stress for the V-Y flap in the release state. The overall trend is quite similar to that in the suturing state but the maximum stress was reduced from 0.038 MPa to 0.021 MPa.

The displacement distribution of the flap in the release state is similar to that in the suturing state. The maximum displacement in the release state is 3.467 mm, which is 1.163 mm lower than that in the suturing state. This might be because the nodes of the V-Y flap model produce a certain amount of contraction in the release state, consistent with actual surgery.

## 4. Discussion

### 4.1. Round Defect and Rectangular Defect

Both round and rectangular shapes are commonly used in V-Y flap surgery. Previous research [12, 13, 15] has systematically studied the flap design and flap advancement of rectangular V-Y flaps. However, for the same lesion, the total area of the defect and flap using round-shaped transplant is smaller than that for rectangular shapes and better postoperative effects have been achieved for the former.

**Figure 7:**
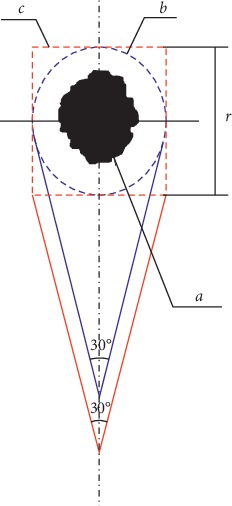
Comparison of two shape defects.

A comparative schematic diagram for the two defect shapes is shown in Figure 7. The black area (a) indicates the lesion area with a circular defect enclosed by the blue dotted curve (b). The red dotted line (c) indicates another rectangular defect plan, and the diameter of the defect is denoted as *r*. The flap apex angle was assumed to be 30°. Thus, the areas of the circular defect and the rectangular defect are expressed as *s*_*w*_^*c*^ and *s*_*w*_^*s*^, the flap areas are *s*_*f*_^*c*^ and *s*_*f*_^*c*^, and the overall concerned surgical areas are *s*^*c*^ and *s*^*s*^, respectively.

According to the cross-constraints,

(14) $$\begin{array}{c} n=\frac{s_{f}^{c}}{s_{w}^{c}}=0.77. \end{array}$$

For round-shaped defect,

(15) $$\begin{array}{c} s^{c}=s_{f}^{c}+s_{w}^{c}=1.77\pi r^{2}. \end{array}$$

For rectangular-shaped defect,

(16) $$\begin{array}{c} s_{w}^{s}=4r^{2}. \end{array}$$

According to the trigonometric function,

(17) $$\begin{array}{c} s_{f}^{s}=r^{2}\cot\left(15^{{}^{\circ}}\right),\\ s^{s}=s_{f}^{s}+s_{w}^{s}=4r^{2}+r^{2}\cot\left(15^{{}^{\circ}}\right). \end{array}$$

The ratio of the rectangular defect to the circular defect is obtained as

(18) $$\begin{array}{c} \frac{s^{s}}{s^{c}}\approx 1.39. \end{array}$$

Then, the surgical area of the rectangular defect is about 1.39 times that of the circular defect for the same lesion.

### 4.2. Position of V-Y Flap Advancement

**Figure 8:**
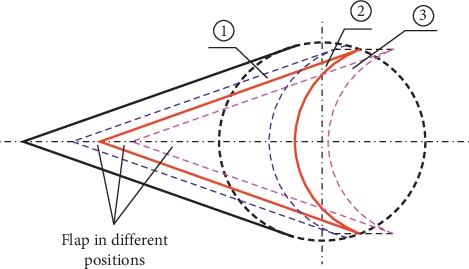
Schematic diagram of flap advancement.

According to clinical observations and experiences, the position of flap advancement impacts the suture effect and three flap advancement plans were evaluated (Figure 8). If the flap is advanced with plan ① (blue dashed line), the arc edge at the bottom of the flap cannot maintain full contact with the edge of the defect. If the flap moves according to plan ③ (purple dashed line), the length of the flap arc edge is longer than the edge of the defect. In both of the abovementioned cases, the flap arc length is not equal to that of the corresponding defect edge and the suturing operation tends to produce skin wrinkles or “cat ears,” which should be avoided in stitching. Thus, only when the flap advances according to plan ②, the flap arc length will equal that of the defect. Theoretically, in this case, flap suturing will not produce any skin wrinkles or “cat ears.”

### 4.3. Finite Element Analysis of the V-Y Flap

V-Y flap advancement causes mechanical creep and elastic stretching of the skin by applying external force to the soft tissue of the skin, resulting in large deformation and large displacement. The authors attempted to achieve the exact distribution of the stress and deformation of the V-Y flap in the suturing and release states, which truly simulates the conditions in V-Y flap surgery. By using nonlinear finite element analysis software, soft tissue suture simulation was performed in this research.

The stress at any point in an elastic object depends entirely on the strain at the time and the position and has nothing to do with the history of the strain. However, for viscoelastic material, the stress at any position depends, not only on the local strain at that time but also the historical strain process. Previous studies on the biomechanical properties of skin materials revealed that mechanical behavior was closely related to the collagen network of skin [21]. Jacquet et al. [22] achieved skin stress-strain curves under different conditions by conducting uniaxial tensile tests on volunteers with different anatomical locations and different directions. Karimi et al. [23] obtained the prelinear viscoelastic properties of rat back and abdominal skin under uniaxial loading.

In handling the boundary conditions, when the V-Y flap was in the suture state, the displacement constraints on the suturing nodes on both sides of the flap were bilaterally symmetrical and no relative displacements occurred between the flap and the whole skin model. Therefore, the outer skin edges must not be restrained but set in a free state which truly simulates real-life V-Y flap situations. As a result, a real V-Y flap deformation model can be obtained. In the release state, the imposed displacements on the suturing nodes are removed and contact conditions are applied. The results of the simulation showed that the flap had good contact with the defect margin and the stitching was uniform and without any deformity. The maximum tension of the flap after suturing was concentrated in the bottom edge and the apex angle of the flap, which was consistent with actual clinical situations. The maximum von Mises stress was 0.021 MPa, which meets the tension limitation of skin, which ranges from 2.5 MPa to 16 MPa [24].

## 5. Conclusion

In this paper, the geometric model of a V-Y flap was established and five key geometric relationships affecting the structure of the V-Y flap were analyzed. A reasonable scheme for a V-Y flap design was proposed by combining previous clinical experience and crossing constraints methods. To achieve the real distribution of stress and displacement of the V-Y flap during surgical operations, the finite element approach was applied to investigate the tension and deformation of the V-Y flap in the suturing and release states. The simulation results verified the rationale of the proposed V-Y flap design method and the preoperative evaluation of the V-Y flap design for clinic practice, which provides a feasible method for clinical V-Y flap design.

## Data Availability

The finite-element model data used to support the findings of this study can be obtained by contacting the first author.
